# Maiden Fair

**Published:** 1890-10

**Authors:** 


### MAIDEN FAIR.

Translated from the German of Henreich Heine, for Hall's Journal of Health.

Maiden fair with lips of cherry,
And with eyes so mild and clear,
Thou art e'en my little fairy,
Ever trusting, ever dear.

Long are now the winter evenings,
Still they all too swiftly go,
At thy side in joyful meetings,
By the hearth's inviting glow.

There to thee my love confessing,
Oft I press thy dainty hand,
E'en by tears the wish expressing,
Only you can understand.

